# Supplementary material for: The importance of shear on the collective charge transport in CDWs revealed by an XFEL source
Source: Sci Adv. 2025 Jan 3;11(1):eadr6034. doi: 10.1126/sciadv.adr6034 (PMC11698093; doi:10.1126/sciadv.adr6034)
Supplement: Supplementary file 1 — Supplementary Text Figs. S1 to S11 References [file sciadv.adr6034_sm.pdf]

Science Advances
AAAS

Supplementary Materials for

The importance of shear on the collective charge transport in CDWs revealed by an XFEL source

David Le Bolloc'h *et al.*

Corresponding author: David Le Bolloc'h, david.le-bolloch@universite-paris-saclay.fr

Sci. Adv. **11**, eadr6034 (2025)
DOI: 10.1126/sciadv.adr6034

This PDF file includes:

Supplementary Text
Figs. S1 to S11
References

Supplementary Text and Figures

(1) Sample and *in situ* transport measurements.

Under an applied external field, incommensurable CDW systems may slide. They exhibit two regimes. Below the threshold field, transport measurements show Ohmic behavior with constant differential resistance. Above threshold, on the other hand, an excess current appears in the sample, directly involving the CDW itself. *In situ* transport measurements clearly show the presence of both regimes in our $NbSe_3$ sample with a threshold field equal to I_S=0.8mA (see the sample image in Fig. S1 and the differential resistance in Fig. S2).

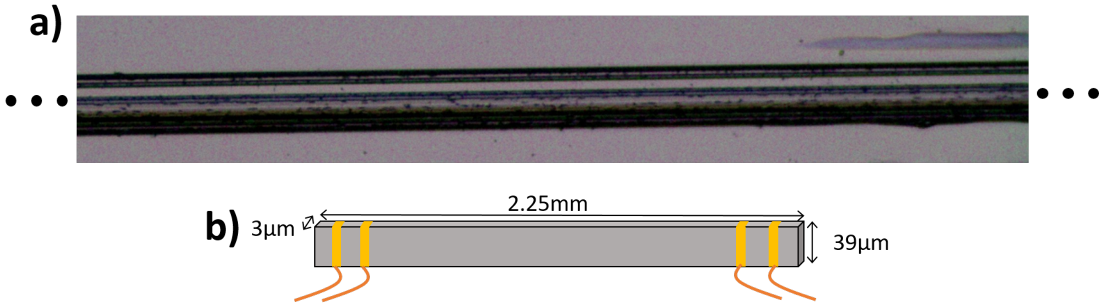

Fig. S1. Sample image. (**A**) Image in the middle of the sample obtained with an optical microscope. The sample size is 39μm $\times$ 3μm $\times$ 2.25mm. A step is visible on the crystal surface. (**B**) Drawing of the sample with its dimensions and electrical contacts in yellow.

(2) Satellite reflections versus applied current.

Fig. S3 shows the square root integrated CDW peak intensity at different currents. This corresponds to the CDW averaged amplitude over the measured region. Variations of less than 5% are observed. Since the average CDW amplitude is weakly affected by the applied current, we consider only phase variations to describe the evolution of the CDW under current.

Let us first discuss the overall evolution of the **2 k**$_F$satellite with applied current. Fig. S4A and Fig. S4B show the peak transverse profiles and Fig. S4C and Fig. S4D show the longitudinal one. The amplitude of variations along the longitudinal q_x direction is much smaller than in transverse direction. The scale in q_x is 4 times larger than in q_y but the shift near threshold

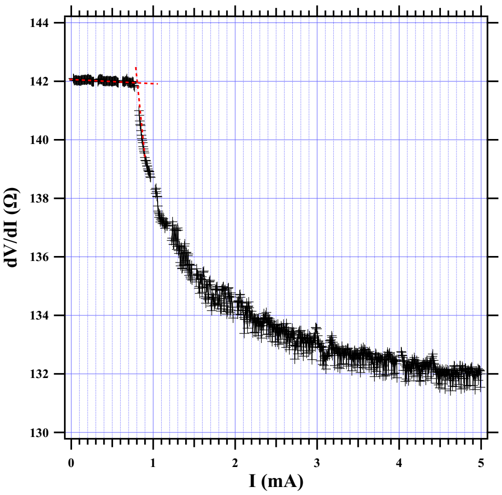

Fig. S2. Transport measurement. Differential resistance measured during the experiment displaying a clear discontinuity at the threshold current I_S=0.8mA.

is nevertheless clearly visible. The peak center of mass (peak position) and standard deviation (proportional to the peak width) are shown in Fig. S5, in both transverse and longitudinal directions. Below threshold, the transverse shift and the transverse width increase with current, reaching a maximum at 1mA (I_S=0.8mA). Above threshold, a decrease of both parameters is observed. We will see later that transverse relaxation above threshold is more clearly observed when considering a quadratic phase model or the genetic code (see also Fig. 5 in the main text).

The longitudinal direction, on the other hand, exhibits a very different behaviour. No change in the longitudinal peak position is observed below threshold. However, an abrupt longitudinal shift is observed at the threshold current, with a saturation above 3mA (see Fig. S5C). This shift in peak position corresponds to a CDW compression, which is compatible with a CDW solitonic charge transport (*34*) where periodic CDW localized 2π phase shifts travels through the sample leading to an increase of the number of CDW wavelengths in the measured region. This CDW compression in the sliding regime, although smaller than close to the electrodes, was also observed far from the electrodes in (*20*). A decreasing standard deviation for current above threshold is observed along the longitudinal direction (see Fig. S5D), similar to the transverse

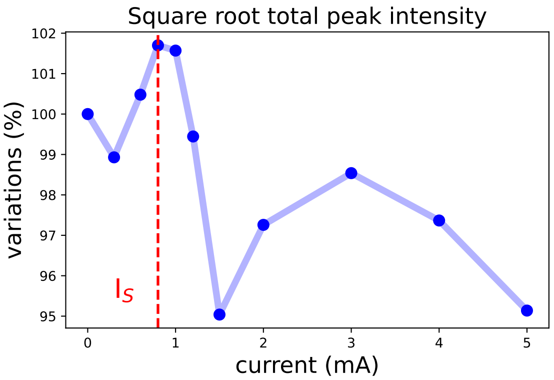

Fig. S3. Integrated intensity. Square root of the integrated peak intensity proportional to the CDW amplitude with less than 5% variations for the different applied currents.

direction but with an amplitude of variation one order of magnitude smaller. This longitudinal peak sharpening in the middle part of the sample was also observed in (*23*) and corresponds to an increase of the longitudinal CDW correlation length above threshold.

(3) Theory of elastic CDW, strongly pinned by sample surfaces and submitted to external electric field

As discussed in the main paper, sliding phenomenon is closely linked to a deformation of the CDW structure. Above threshold, a contraction and a dilatation of the CDW period at the two electrical contacts is observed (*20,23*). Below threshold, a shear effect in the middle part of the sample has been observed in (*25*) and in the present paper. In both cases, the two types of deformation, longitudinal and transverse, extend over a very long distance. Shear deformation, which leads to wavefront curvature, extends over several tens of micrometers, more than 4 orders of magnitude larger than the CDW wavelength ($\lambda_F = 14\mathring{A}$).

The two types of deformations, in the elastic regime below threshold, can be reproduced by considering the CDW as an elastic object, strongly pinned by sample surfaces (*26*). The CDW is described as a periodic charge modulation associated to a periodic lattice distortion, $\rho(\vec{r}) = A(\vec{r})\cos(2k_F x + \phi(\vec{r}))$, where $A(\vec{r})$ and $\phi(\vec{r})$ are respectively the CDW amplitude

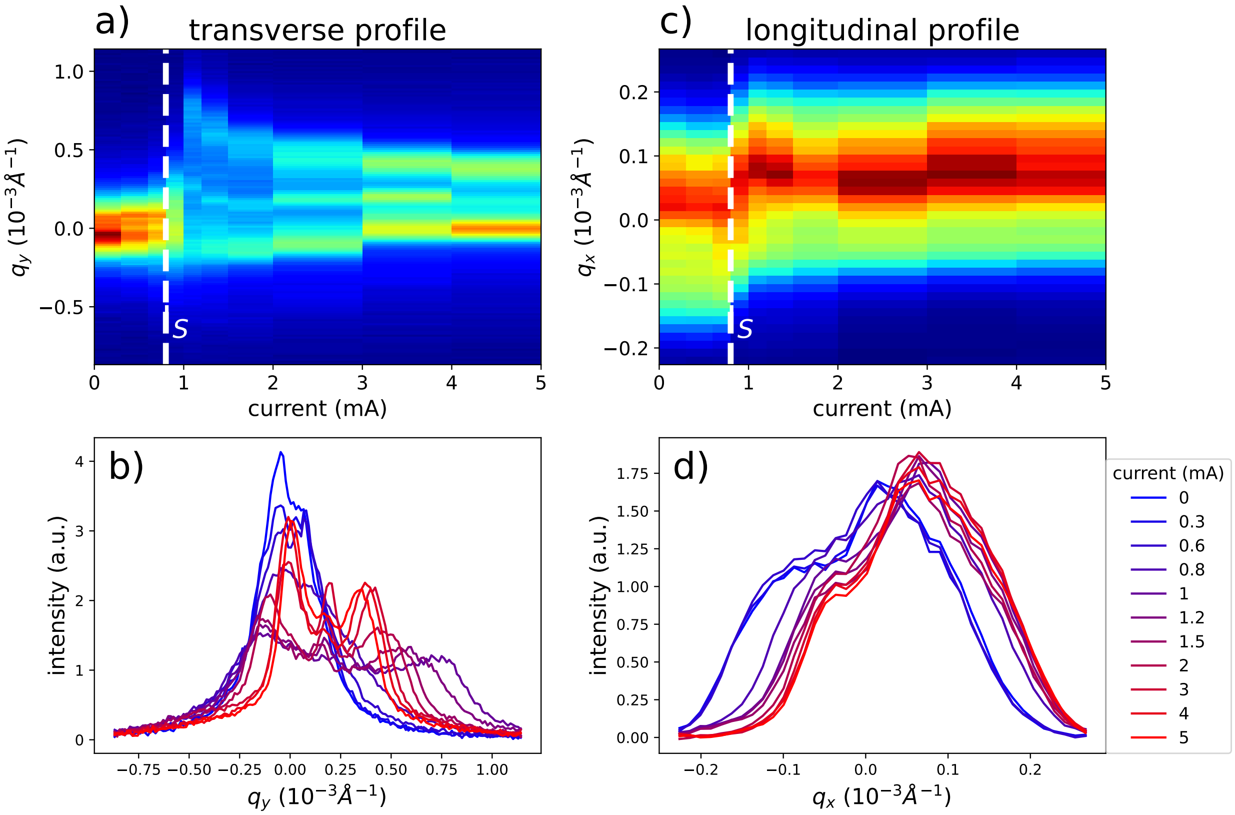

Fig. S4. Diffraction profiles. $\mathbf{2k}_F$ satellite profile as a function of applied currents. (**A-B**) Transverse peak profile at different currents and (**C-D**) longitudinal peak profile. We emphasize that the range along the longitudinal q_x direction is smaller than along the transverse q_y, showing that the longitudinal variations, in position and in width, are much smaller than along the transverse one.

and the CDW phase with spatial dependence. We only consider a phase model and an applied electric field with component E along the $\mathbf{2k}_F$ direction, parallel to the x axis. The CDW behavior can be described by the following free energy:

$$\mathcal{F}[\phi] \propto \int_{\mathcal{V}} d^3\vec{r} \left\{ C^{ij}\phi_i\phi_j + V_{imp}(\phi) - \eta E x \phi_x \right\} \tag{1}$$

$$\phi_i = \partial_i\phi, \qquad \vec{r} \in \mathcal{V} \equiv \left\{ \vec{r} \in R^3 \text{ with } x_i \le L_i/2 \right\} \tag{2}$$

where $i, j = x, y, z$, $C^{ij} = c_i c_j \delta^{ij}$ with c_x, c_y, c_z being the CDW longitudinal and transverse elastic coefficients, L_x is the contact distance, L_y and L_z are the transverse sample lengths. We

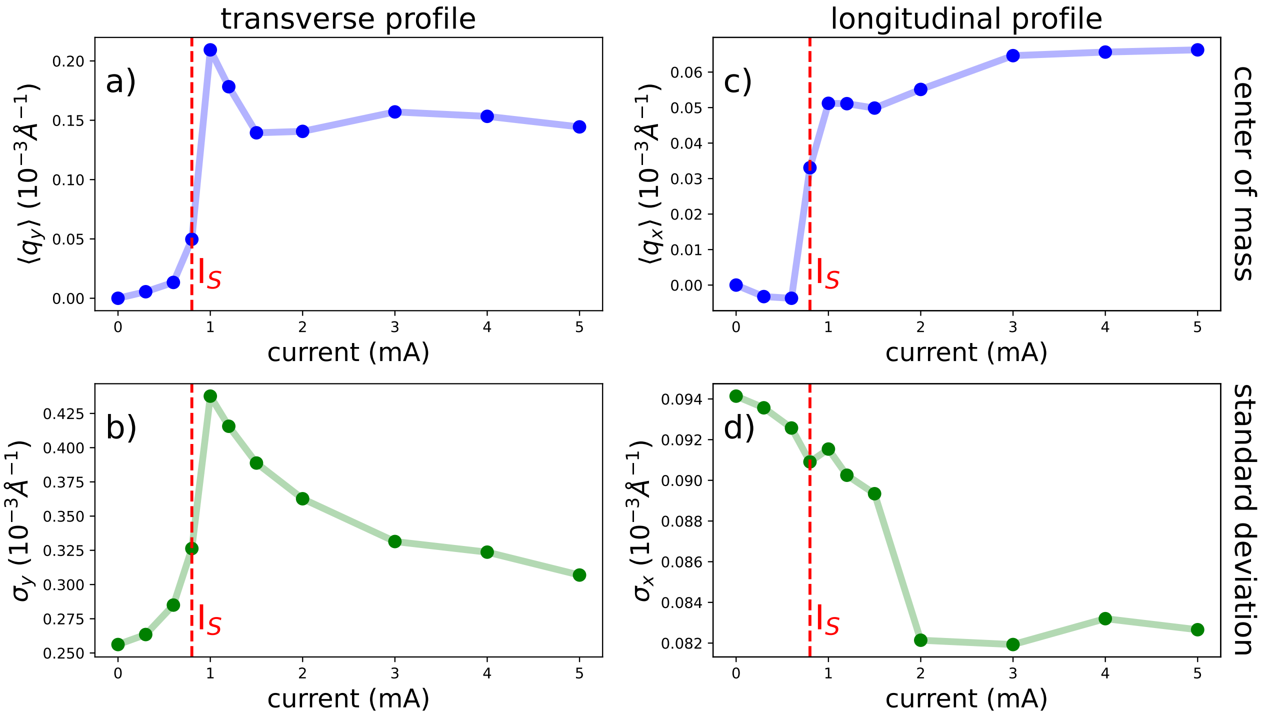

Fig. S5. Transverse and longitudinal behavior with current. 2k$_F$ satellite profile as a function of applied currents. (**A**) Center of mass along the transverse direction showing a global shift near the threshold current I_S=0.8mA. (**B**) Transverse profile standard deviation. The peak width reaches a maximum at 1mA. (**C**) Center of mass along the longitudinal direction and (**D**) the longitudinal standard deviation. We emphasize that the range in q_x and in σ_x are smaller than q_y and σ_y.

choose to consider a bulk impurity pinning potential $V_{imp}(\phi) \equiv \omega_0^2[1-\cos(\phi)]$ with the pinning frequency ω_0 (*35,36*) . The last term corresponds to the interaction between the CDW and the applied electric field coupling the longitudinal gradient ϕ_x and the applied electric potential Ex where η is a temperature dependent coupling coefficient (*37*) . Minimizing $\mathcal{F}[\phi]$ gives:

$$2\left(c_x^2\phi_{xx} + c_y^2\phi_{yy} + c_z^2\phi_{zz}\right) - \omega_0^2\phi \approx \eta E \tag{3}$$

In that case, the second-order equation (Eq.3) has to be solved with boundary conditions by setting the phase to zero on all surfaces:

$$\phi(\vec{r}) = 0, \qquad \forall \vec{r} \in \partial\mathcal{V}. \tag{4}$$

By rescaling $x_j = c_j\sqrt{\frac{2}{\eta}}x'_j$, $L_j = c_j\sqrt{\frac{2}{\eta}}L'_j$ and $\omega^2 = \frac{\omega_0^2}{\eta}$, the phase equation Eq.3 become:

$$\left(\Delta' - \omega^2\right)\phi = E \tag{5}$$

with the Dirichlet conditions:

$$\phi(\vec{r'}) = 0, \qquad \forall \vec{r'} \in \partial\mathcal{V}' \tag{6}$$

where $\Delta' = \frac{\partial^2}{\partial x'^2} + \frac{\partial^2}{\partial y'^2} + \frac{\partial^2}{\partial z'^2}$ is the rescaled Laplacian operator. Eq.5 is the screened Pois-

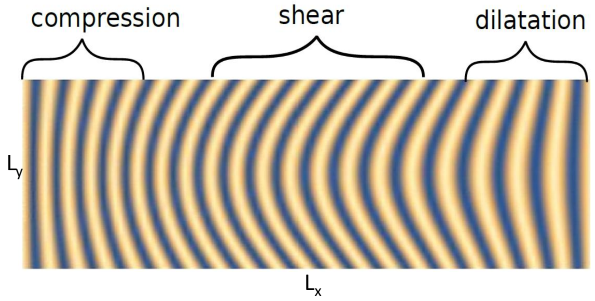

Fig. S6. Quadratic pinned model: analytical first order solution. CDW under electric field by considering surface pinning leading to extended CDW deformations through the entire crystal with a compression and dilatation at the electrodes and bent wavefronts in the central part. For clarity, the CDW period is considerably increased compared to the sample size. In reality, the CDW wavelength in $NbSe_3$ is $\lambda_F = 14\mathring{A}$, while the typical sample width is around 40μm.

son equation whose solution can be obtained by using the Green function and image charge method (*38*). The exact solution takes the form of an infinite sum (*26*) that converges rapidly so that keeping the first term is a good approximation (the first-order approximation is a good approximation especially when $\frac{L_x}{c_x} \sim \frac{L_y}{c_y} \sim \frac{L_z}{c_z}$ and $\omega_0 < 1$). The first-order expression reads:

$$\phi(\vec{r}) \propto -E\eta\beta\cos(\pi\frac{x}{L_x})\cos(\pi\frac{y}{L_y})\cos(\pi\frac{z}{L_z}) \tag{7}$$

with the sample-size dependent parameter β:

$$\beta = \frac{1}{\frac{c_x^2}{L_x^2} + \frac{c_y^2}{L_y^2} + \frac{c_z^2}{L_z^2} + \frac{\omega_0^2}{2\pi^2}}$$

The Dirichlet conditions are well fulfilled in Eq.7 ($\phi(\pm L_x/2, y, z) = \phi(x, \pm L_y/2, z) = \phi(x, y, \pm L_z/2)$=0). At the first order, the CDW phase $\phi(\vec{r})$ is proportional to a simple product of cosine functions leading to a CDW compression and dilatation at both ends and a shear in the central part of the sample. The typical CDW deformation under external current is displayed in Fig. S6. This static model is not intended to describe the non-linearity observed above threshold field, but correctly describes the increase in curvature observed at the center of the sample, considering a weak bulk frequency pinning ω_0 (*25*).

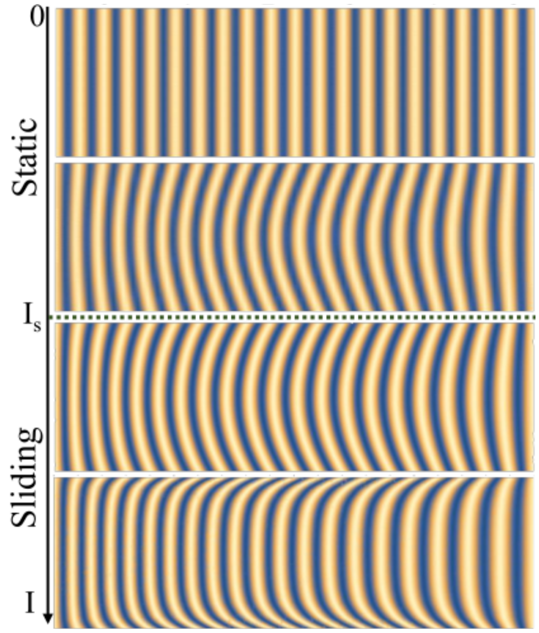

Fig. S7. Quadratic pinned model: numerical solution. CDW deformation obtained by numerically solving Eq.3 with $\omega_0 = c_z = 0$ and by decreasing the ratio c_y/c_x with current. Although this static model is phenomenological, it does reproduce the overall situation observed here, with an increasing compression and a decrease in curvature between two pinning centers above threshold.

(4) Diffraction pattern interpreted through a quadratic phase.

Let's now consider the case where the evolution of diffraction patterns is mainly due to wavefront curvature. A good approximation to describe the diffraction patterns is to consider a quadratic phase, proportional to y^2, to account for bent wavefronts, plus an additional linear y

dependent term to account for wavefront tilt:

$$\phi(y) \equiv \alpha y^2 + \beta y \tag{8}$$

where y is the transverse position, α and β are fitting parameters. Assuming a constant CDW amplitude, the corresponding diffracted intensity around the CDW satellite is given by:

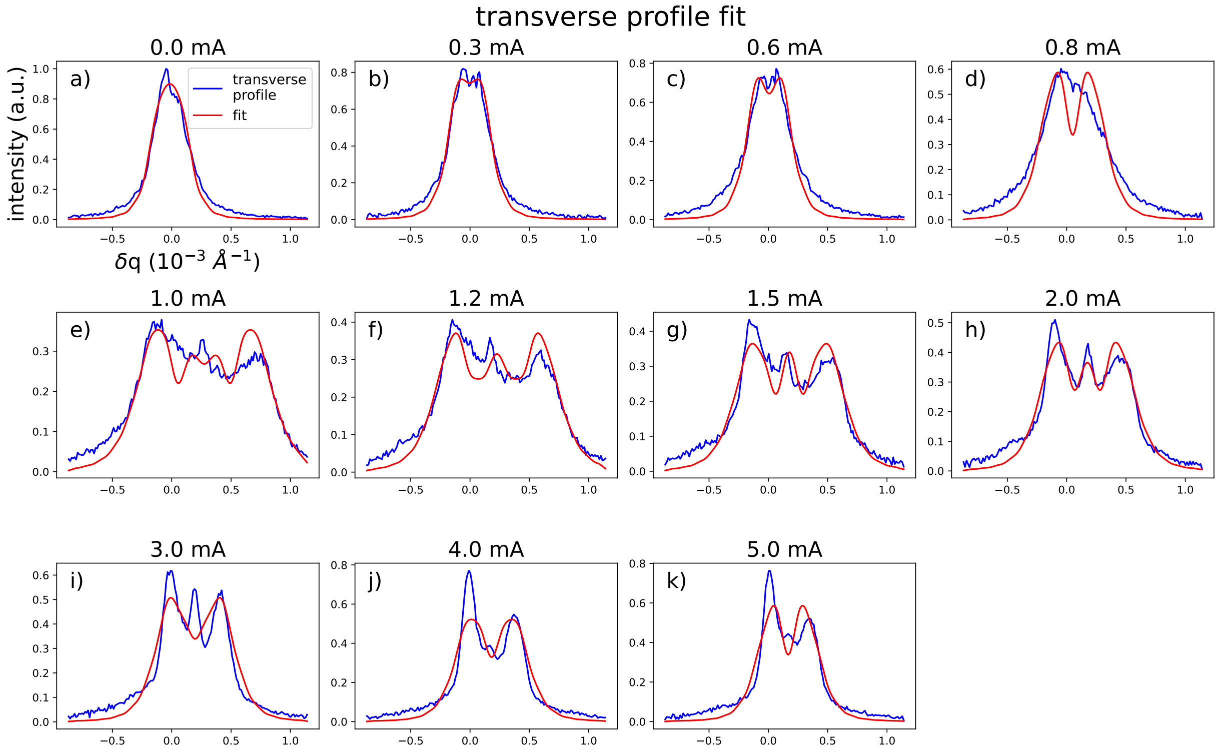

Fig. S8. Fit from a quadratic phase. Transverse profile fits considering a quadratic CDW phase and a tilt.

$$I(q) \propto \left| \int_a^b e^{iqy} e^{i\phi(y)} \, dy \right|^2 \tag{9}$$

where a nd b are the beam transverse limits (since the x-ray beam is smaller than the sample width), q is the transverse wavevector. The analytical solution reads :

$$I(q) = A \left| \text{erf}\left[e^{i\pi 4} \sqrt{(\alpha)} \left(b + \frac{q+\beta}{2\alpha} \right) \right] - \text{erf}\left[e^{i\pi 4} \sqrt{(\alpha)} \left(a + \frac{q+\beta}{2\alpha} \right) \right] \right|^2 \tag{10}$$

where A is an overall factor and erf is the complex error function defined by

$$\mathrm{erf}(z) = \frac{2}{\sqrt{\pi}} \int_0^z e^{-t^2} dt \tag{11}$$

In order to take into account beam pointing errors and intensity fluctuations, an additional

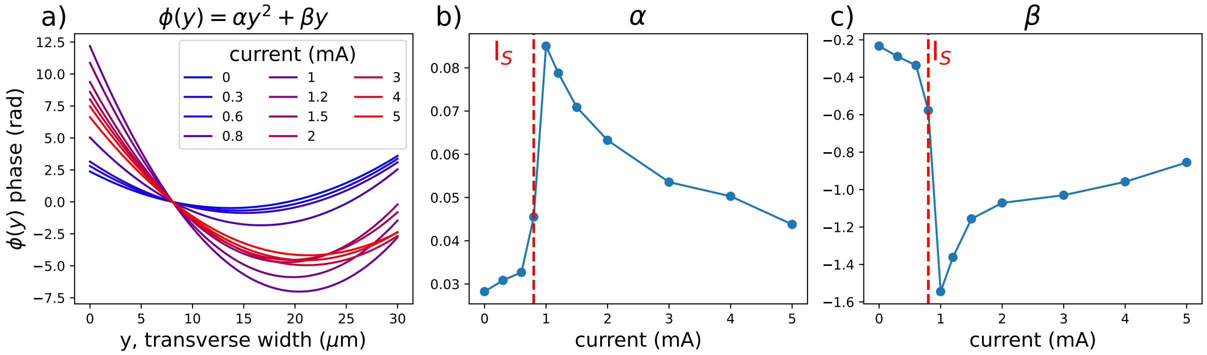

Fig. S9. Parameters obtained with the quadratic phase model. (**A**) Transverse phase obtained after fitting the experimental profiles and by considering a quadratic phase profile: $\phi(y) = \alpha y^2 + \beta y$. (**B**) Variation of α and (**C**) β with applied current. The amplitude of CDW wavefront curvature reaches a maximum at 1mA, then start decreasing back at higher currents.

Lorentzian convolution with a free width fitting parameter has been added to fit the experimental profiles. This Lorentzian width is kept constant between all current during the fitting procedure. The resulting fits are shown in Fig. S8. As we can see, despite the reduced number of parameters, taking into account bent CDW wavefronts, roughly reproduces the global behavior of the diffraction profiles with current. The corresponding parameters with currents are shown in Fig. S9. The increase of the CDW curvature α with increasing current is still observed until 1mA (see Fig. S9B). Above 1 mA, a strong decrease is observed. This threshold effect is visible for the α and β parameters (Fig. S9B and Fig. S9C). However, as visible in Fig. S8, a simple quadratic CDW phase can not reproduce the measured peak asymmetry.

(5) Phase retrieval using genetic code

As explained in the main text, we used a genetic code to retrieve the CDW phase more precisely, without neglecting the asymmetry of peak profiles. The most complex part of the code

is the calculation of profiles (involving a Fourier transform and convolution) for a population of trial vectors, at each iteration of the DE algorithm. This part was coded in OpenCL and run on the NVIDIA RTX 3060 GPU, enabling 700 thousand test configurations per second.

The phase was expanded on a Fourier basis closely related to the elastic pinned model discussed previously, with the expected cosine function as the first term:

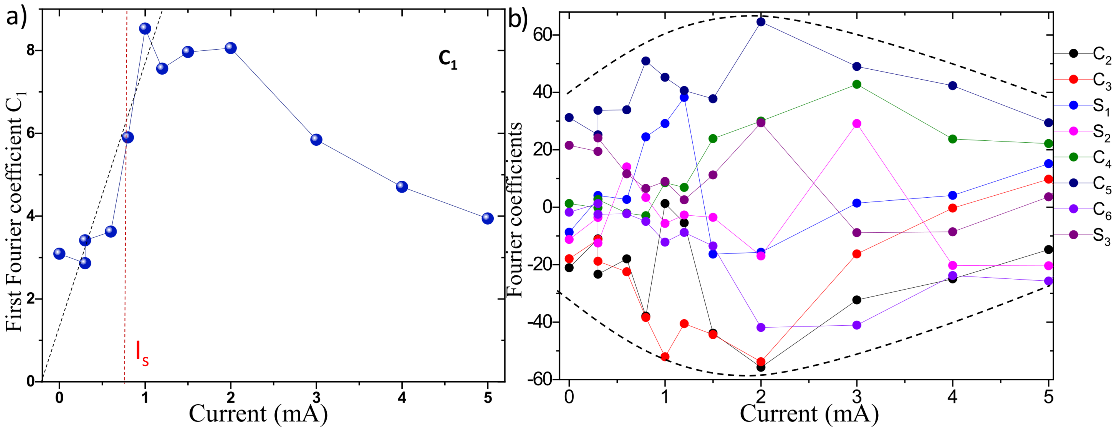

Fig. S10. Components of Fourier series versus current. (**A**) First Fourier coefficient and (**B**) the higher order terms as a function of current. The overall envelope of coefficients increases above threshold and decreases for larger currents (see dotted lines).

$$\phi(y) = c_1 \cos(\pi \frac{y}{L_y}) + \sum_{n=2}^{6} [c_n \cos(n\pi \frac{y}{L_y}) + s_n \sin(n\pi \frac{y}{L_y})] + \alpha y \qquad (12)$$

with $y \in [-L_y/2, L_y/2]$. The first coefficient c_1 is nothing else but the effective electric field E_e felt by the CDW in the previous model. The slope α is the way to take into account the shift observed along the vertical direction of the camera (see Fig. 2A in the main text), and without considering too many sinus terms in the series. The actual number of terms required in the series was determined by trying different fits with an increasing number of terms in the series until no substantial improvement was found. Note that all fits were run several times with different initial coefficient values, to check that the procedure always converges to the same phase $\phi(y)$. In addition to the phase expansion, the diffraction pattern results from a convolution. Two Lorentzian have been used as a resolution function to take into account the extended feet of

profiles. A tiny slope in the background is also used. Finally, 14 free parameters have been considered. The evolution of the Fourier coefficients appearing in Eq.12 are displayed in Fig. S10B versus current. Despite the number of parameters and the few constraints imposed on them, the components show a relatively smooth evolution.

(6) Transverse profile with currents: different methods

Several methods can be used to follow the CDW average shear deformation at different currents. In this section, we show 4 different methods showing the same trend.

The 1st method shown in Fig. S11A is the peak standard deviation along the transverse direction which is proportional to the peak transverse width evolution.

The 2nd method shown in Fig. S11B takes the CDW phase reconstructed from the transverse fit shown in F ig. 3B of the main t ext. We then proceed to compute the second order gradient and take the averaged absolute value.

The 3rd method is similar to the previous one, but we instead make a linear fit of the phase derivative shown in Fig. 3C of the main text. The slope calculated from this fit is then shown in absolute value in Sup. Fig. S11C.

Finally, our 4th method shown in Sup. Fig. S11D consists in plotting the c_1 fitted coefficient from Eq12.

Despite slight variations, the same trend is observed for each method with a strong increase in CDW shear up to 1mA. This shear effect then decreases at higher currents due to a CDW relaxation in the transverse direction.

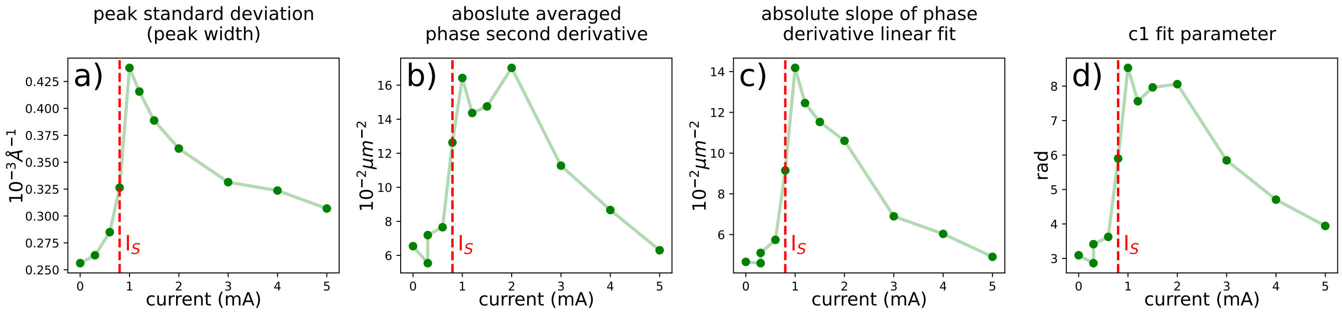

Fig. S11. Transverse shear verus current. (**A**) Peak transverse profile standard deviation. (**B**) Average of the fitted phase second derivative (absolute value). (**C**) Slope from the linear fit of the phase derivative shown in Fig. 3 of the main text. (**D**) c_1 parameter from Eq12. The threshold current I_S=0.8mA position is shown with a borken red line.

REFERENCES AND NOTES

1. A. H. Moudden, J. D. Axe, P. Monceau, F. Levy, q_1 charge-density wave in $NbSe_3$. *Phys. Rev. Lett.* **65**, 223–226 (1990).

2. A. Singer, S. K. K. Patel, R. Kukreja, V. Uhlir, J. Wingert, S. Festersen, D. Zhu, J. M. Glownia, H. T. Lemke, S. Nelson, M. Kozina, K. Rossnagel, M. Bauer, B. M. Murphy, O. M. Magnussen, E. E. Fullerton, O. G. Shpyrko, Photoinduced enhancement of the charge density wave amplitude. *Phys. Rev. Lett.* **117**, 056401 (2016).

3. V. L. R. Jacques, C. Laulhé, N. Moisan, S. Ravy, D. Le Bolloc'h, Laser-induced charge-density-wave transient depinning in chromium. *Phys. Rev. Lett.* **117**, 156401 (2016).

4. A. Zong, P. E. Dolgirev, A. Kogar, E. Ergeçen, M. B. Yilmaz, Y.-Q. Bie, T. Rohwer, I-C. Tung, J. Straquadine, X. Wang, Y. Yang, X. Shen, R. Li, J. Yang, S. Park, M. C. Hoffmann, B. K. Ofori-Okai, M. E. Kozina, H. Wen, X. Wang, I. R. Fisher, P. Jarillo-Herrero, N. Gedik, Dynamical slowing-down in an ultrafast photoinduced phase transition. *Phys. Rev. Lett.* **123**, 097601 (2019).

5. R. V. Yusupov, T. Mertelj, J.-H. Chu, I. R. Fisher, D. Mihailovic, Single-particle and collective mode couplings associated with 1- and 2-directional electronic ordering in metallic $R\mathrm{Te}_3$(R = Ho, Dy, Tb). *Phys. Rev. Lett.* **101**, 246402 (2008).

6. I. Gonzalez-Vallejo, V. L. R. Jacques, D. Boschetto, G. Rizza, A. Hadj-Azzem, J. Faure, D. Le Bolloc'h, Time-resolved structural dynamics of the out-of-equilibrium charge density wave phase transition in $GdTe_3$. *Struct. Dyn.* **9**, 014502 (2022).

7. F. Schmitt, P. S. Kirchmann, U. Bovensiepen, R. G. Moore, L. Rettig, M. Krenz, J.-H. Chu, N. Ru, L. Perfetti, D. H. Lu, M. Wolf, I. R. Fisher, Z.-X. Shen, Transient electronic structure and melting of a charge density wave in $TbTe_3$. *Science* **321**, 1649 (2008).

8. A. Kogar, A. Zong, P. E. Dolgirev, X. Shen, J. Straquadine, Y.-Q. Bie, X. Wang, T. Rohwer, I-C. Tung, Y. Yang, R. Li, J. Yang, S. Weathersby, S. Park, M. E. Kozina, E. J. Sie, H. Wen, P.

Jarillo-Herrero, I. R. Fisher, X. Wang, N. Gedik, Light-induced charge density wave in $LaTe_3$. *Nat. Phys.* **16**, 159–163 (2020).

9. D. A. Zocco, J. J. Hamlin, K. Grube, J.-H. Chu, H.-H. Kuo, I. R. Fisher, M. B. Maple, Pressure dependence of the charge-density-wave and superconducting states in $GdTe_3$, $TbTe_3$, and $DyTe_3$. *Phys. Rev. B* **91**, 205114 (2015).

10. N. Ru, C. L. Condron, G. Y. Margulis, K. Y. Shin, J. Laverock, S. B. Dugdale, M. F. Toney, I. R. Fisher, Effect of chemical pressure on the charge density wave transition in rare-earth tritellurides RTe$_3$. *Phys. Rev. B* **77**, 249908 (2008).

11. J. A. W. Straquadine, M. S. Ikeda, I. R. Fisher, Evidence for realignment of the charge density wave state in $ErTe_3$ and $TmTe_3$ under uniaxial stress via elastocaloric and elastoresistivity measurements. *Phys. Rev. X* **12**, 021046 (2022).

12. A. Gallo-Frantz, V. L. R. Jacques, A. A. Sinchenko, D. Ghoneim, L. Ortega, P. Godard, P.-O. Renault, A. Hadj-Azzem, J. E. Lorenzo, P. Monceau, D. Thiaudière, P. D. Grigoriev, E. Bellec, D. Le Bolloc'h, Charge density waves tuned by biaxial tensile stress. *Nat. Comm.* **15**, 3667 (2024).

13. P. Monceau, J. Richard, M. Renard, Interference effects of the charge-density-wave motion in $NbSe_3$. *Phys. Rev. Lett.* **45**, 43 (1980).

14. R. M. Fleming, C. C. Grimes, Sliding-mode conductivity in $NbSe_3$: Observation of a threshold electric field and conduction noise. *Phys. Rev. Lett.* **42**, 1423 (1979).

15. D. Le Bolloc'h, A. A. Sinchenko, V. L. R. Jacques, L. Ortega, J. E. Lorenzo, G. A. Chahine, P. Lejay, P. Monceau, Effect of dimensionality on sliding charge density waves: The quasi-two-dimensional $TbTe_3$ system probed by coherent x-ray diffraction. *Phys. Rev. B* **93**, 165124 (2016).

16. D. Le Bolloc'h, E. Bellec, N. Kirova, V. Jacques, Tracking defects of electronic crystals by coherent x-ray diffraction. *Symmetry* **15**, 1449 (2023).

17. D. DiCarlo, E. Sweetland, M. Sutton, J. D. Brock, R. E. Thorne, Field-induced charge-density-wave deformations and phase slip in $NbSe_3$. *Phys. Rev. Lett.* **70**, 845 (1993).

18. H. Requardt, F. Y. Nad, P. Monceau, R. Currat, J. E. Lorenzo, S. Brazovskii, N. Kirova, G. Grübel, C. Vettier, Direct observation of charge density wave current conversion by spatially resolved synchrotron x-ray studies in $NbSe_3$. *Phys. Rev. Lett.* **80**, 5631 (1998).

19. N. P. Ong, K. Maki, Generation of charge-density-wave conduction noise by moving phase vortices. *Phys. Rev. B* **32**, 6582 (1985).

20. S. Brazovskii, N. Kirova, H. Requardt, F. Y. Nad, P. Monceau, R. Currat, J. E. Lorenzo, G. Grübel, C. Vettier, Plastic sliding of charge density waves: X-ray space resolved-studies versus theory of current conversion. *Phys. Rev. B* **61**, 10640 (2000).

21. Y. Li, S. G. Lemay, J. H. Price, K. Cicak, K. O'Neill, K. Ringland, K. D. Finkelstein, J. D. Brock, R. E. Thorne, Imaging shear in sliding charge-density waves by x-ray diffraction topography. *Phys. Rev. Lett.* **83**, 3514 (1999).

22. E. Bellec, I. Gonzalez-Vallejo, V. L. R. Jacques, A. A. Sinchenko, A. P. Orlov, P. Monceau, S. J. Leake, D. Le Bolloc'h, Evidence of charge density wave transverse pinning by x-ray microdiffraction. *Phys. Rev. B* **101**, 125122 (2020).

23. R. Alonso-Mori, C. Caronna, M. Chollet, R. Curtis, D. S. Damiani, J. Defever, Y. Feng, D. L. Flath, J. M. Glownia, S. Lee, H. T. Lemke, S. Nelson, E. Bong, M. Sikorski, S. Song, V. Srinivasan, D. Stefanescu, D. Zhu, A. Robert, The x-ray correlation spectroscopy instrument at the Linac Coherent Light Source. *J. Synchrotron Radiat.* **22**, 508–513 (2015).

24. Materials and Methods are available as Supplementary Materials.

25. R. Danneau, A. Ayari, D. Rideau, H. Requardt, J. E. Lorenzo, L. Ortega, P. Monceau, R. Currat, G. Grübel, Motional ordering of a charge-density wave in the sliding state. *Phys. Rev. Lett.* **89**, 106404 (2002).

26. E. Bellec, V. Jacques, J. Caillaux, D. Le Bolloch, The essential role of surface pinning in the dynamics of charge density waves submitted to external dc fields. *Eur. Phys. J. B.* **93**, 165 (2020).

27. D. Feinberg, J. Friedel, Elastic and plastic deformations of charge density waves. *J. Physique* **49**, 485–496 (1988)

28. C. Kim, V. Chamard, J. Hallmann, T. Roth, W. Lu, U. Boesenberg, A. Zozulya, S. Leake, A. Madsen, Three-dimensional imaging of phase ordering in an Fe-Al alloy by bragg ptychography. *Phys. Rev. Lett.* **121**, 256101 (2018)

29. R. Storn, K. Price, Differential evolution – A simple and efficient heuristic for global optimization over continuous spaces. *J. Glob. Optim.* **11**, 341–359 (1997).

30. M. Wormington, C. Panaccione, K. M. Matney, D. K. Bowen, Characterization of structures from X-ray scattering data using genetic algorithms. *Phil. Trans. R. Soc. A Math. Phys. Eng. Sci.* 10.1098/rsta.1999.0469 (1999).

31. S. Das, P. N. Suganthan, Differential evolution: A survey of the state-of-the-art. *IEEE Trans. Evol. Comput.* **15**, 4–31 (2011).

32. A. Zettl, G. Grüner, Onset of charge-density-wave conduction: Switching and hysteresis in $NbSe_3$. *Phys. Rev. B* **26**, 2298 (1982).

33. E. Pinsolle, N. Kirova, V. Jacques, A. Sinchenko, D. Le Bolloc'h, Creep, flow, and phase slippage regimes: An extensive view of the sliding charge-density wave revealed by coherent x-ray diffraction. *Phys. Rev. Lett.* **109**, 256402 (2012).

34. A. Rojo-Bravo, V. L. R. Jacques, D. Le Bolloc'h, Collective transport of charges in charge density wave systems based on traveling soliton lattices. *Phys. Rev. B* **94**, 201120 (2016).

35. H. Fukuyama, P. A. Lee, Dynamics of the charge-density wave. I. Impurity pinning in a single chain. *Phys. Rev. B* **17**, 535 (1978).

36. G. Grüner, *Density Waves in Solids*, Advanced book program: Addison-Wesley (Perseus Books Group, 2000).

37. M. Hayashi, H. Yoshioka, On the Ginzburg-Landau free energy of charge density waves with a three-dimensional order. arXiv:cond-mat/0010102 (2000).

38. K. F. Riley, M. P. Hobson, S. J. Bence, *Mathematical Methods for Physics and Engineering: A Comprehensive Guide* (Cambridge Univ. Press, 2nd ed. 2002).
